# An Update of Cutaneous Melanoma Patients Treated in Adjuvancy With the Allogeneic Melanoma Vaccine VACCIMEL and Presentation of a Selected Case Report With In-Transit Metastases

**DOI:** 10.3389/fimmu.2022.842555

**Published:** 2022-04-01

**Authors:** Ana Mordoh, Mariana Aris, Ibel Carri, Alicia Inés Bravo, Enrique Podaza, Juan Carlos Triviño Pardo, Gerardo Rubén Cueto, María Marcela Barrio, José Mordoh

**Affiliations:** ^1^ División Dermatología, Hospital de Clínicas, Buenos Aires, Argentina; ^2^ Centro de Investigaciones Oncológicas Fundación Cáncer, Buenos Aires, Argentina; ^3^ Laboratorio de Inmunoinformática y Machine Learning, Instituto de Investigaciones Biotecnológicas, Universidad Nacional de San Martín, Buenos Aires, Argentina; ^4^ Laboratorio de Cancerología, Fundación Instituto Leloir, Buenos Aires, Argentina; ^5^ Englander Institute for Precision Medicine, Weill Cornell Medicine, New York, NY, United States; ^6^ Unidad de Bioinformática, Sistemas Genómicos Grupo Biomédico ASCIRES, Valencia, Spain; ^7^ Grupo de Bioestadística Aplicada, Facultad de Ciencias Exactas y Naturales, Universidad de Buenos Aires, Buenos Aires, Argentina; ^8^ Departamento de Bioterapia, Instituto Alexander Fleming, Buenos Aires, Argentina

**Keywords:** cutaneous melanoma (CM), VACCIMEL, therapeutic vaccine, adjuvancy, immune checkpoint inhibitor (ICKi), Bacille Calmette-Guérin (BCG)

## Abstract

The CSF-470 vaccine (VACCIMEL) plus BCG and GM-CSF as adjuvants has been assayed in cutaneous melanoma patients. In the adjuvant randomized Phase II study CASVAC-0401, vaccinated patients had longer distant metastasis-free survival (DMFS) than those treated with IFNα2b. Five years after locking the data, an actualization was performed. The benefit in DMFS was maintained in the vaccinated group versus the IFNα2b-treated group (*p* = 0.035), with a median DMFS of 96 months for VACCIMEL and 13 months for IFNα2b. The favorable risk–benefit ratio was maintained. DMFS was also analyzed as a single cohort in all the IIB, IIC, and III patients (*n* = 30) who had been treated with VACCIMEL. The median DMFS was 169 months, and at 48 months follow-up, it was 71.4%, which was not statistically different from DMFS of previously published results obtained in adjuvancy with ipilimumab, pembrolizumab, nivolumab, or dabrafenib/trametinib. The possible toxicity of combining VACCIMEL with anti-immune checkpoint inhibitors (ICKi) was analyzed, especially since VACCIMEL was co-adjuvated with BCG in every vaccination. A patient with in-transit metastases was studied to produce a proof of concept. During treatment with VACCIMEL, the patient developed T-cell clones reactive towards tumor-associated antigens. Three years after ending the VACCIMEL study, the patient progressed and was treated with ICKi. During ICKi treatment, the patient did not reveal any toxicity due to previous BCG treatment. When she recurred after a 4-year treatment with nivolumab, a biopsy was obtained and immunohistochemistry and RNA-seq were performed. The tumor maintained expression of tumor-associated antigens and HLA-I and immune infiltration, with immunoreactive and immunosuppressive features. VACCIMEL plus BCG and GM-CSF is an effective treatment in adjuvancy for stages IIB, IIC, and III cutaneous melanoma patients, and it is compatible with subsequent treatments with ICKi.

## Introduction

Cutaneous melanoma (CM) is one of the most mutated and immunogenic tumors (1, 2). Besides immune reactivity against non-mutated antigens, exemplified by diseases such as vitiligo (3), the high mutation rate generates neoantigens (neoAgs), peculiar to each patient. Such neoAgs, derived from somatic mutations and therefore previously undetected by the immune system, may elicit immune responses. Anti-immune checkpoint monoclonal antibodies (ICKi) are established therapies both in advanced CM and in adjuvancy (4–6). However, they are only effective against a fraction of patients. It has been consistently shown in several trials of adjuvant immunotherapies, that approximately 50% of stage III CM patients do not spontaneously progress despite not receiving any treatment (trials’ placebo arms), and that ICKi only add protection to about 10%–15% of the total population (4–6). A possible cause of the lack of response in some patients could be a shortage in the pre-treatment spontaneous availability of tumor-targeted CD4 and CD8 lymphocytes. Therefore, a re-emerging approach is the use of therapeutic vaccination to increase the number and quality of effector T lymphocytes. Such vaccines may use modified RNA encoding for tumor-associated antigens (TAA) (7) or neoAgs (8). We have developed a multi-antigenic vaccine, consisting of a mixture of irradiated allogeneic melanoma cell lines (VACCIMEL), adjuvated with BCG and GM-CSF. In a Phase I study, we observed that the toxicity of the combination was minimal and that patients from stages IIB and III had long intervals without relapse (9). Subsequently, we performed the CASVAC-0401 randomized, adjuvant Phase II study in CM patients stages IIB, IIC, and III, comparing the CSF-470 vaccine (hereafter VACCIMEL) plus BCG and GM-CSF versus medium dose IFNα2b. We demonstrated that vaccinated patients had a longer distant metastasis-free survival (DMFS) than those treated with IFNα2b, and that cellular immunity was triggered (10). In order to establish the durability of the responses, we have updated the clinical results 5 years after closing the data collection of the Phase II CASVAC-0401 study.

In ulterior studies, we have conclusively demonstrated that vaccination triggered cellular immunity to previously unrecognized shared TAA and to the own patients´ tumor neoAgs (11). We also analyzed the TCRβ repertoire present at the vaccination sites, metastasis, and blood, and demonstrated that an expansion of TCRβ clonotypes occurred after vaccination, some of which were shared among the vaccinal site, skin metastases, and peripheral blood (12) (13). In view of the previously mentioned evidence, it seems plausible that VACCIMEL could provide a broader range of CD4 and CD8 clonotypes to enhance efficacy of ICKi. This appears to be the case, since 3/3 vaccinated patients who progressed to metastatic disease and were treated with ICKi achieved complete responses without added toxicities (JM, personal communication).

Since VACCIMEL is administered with live *Bacillus Calmette-Guerin* (BCG) in every vaccination, the safety of the sequential combination had to be demonstrated. The work of Finnefrock et al. demonstrated that in *rhesus macaques*, the administration of anti-PD-1 monoclonal antibodies (mAbs) induced a transitory reactivation of SIV in chronically infected animals (14). This result probably led to prohibit the administration of live attenuated vaccines in clinical trials of ICKi (15, 16). However, recent data demonstrated that in cancer patients with HIV, HBV, or HCV infection and treated with ICKi, the safety and efficacy profile of therapy were similar to those patients without chronic viral illness (17). As it refers to BCG, and as a proof of concept on the safety of combining BCG–containing vaccines with ulterior ICKi treatment, we shall present here the case of a patient with in-transit metastases (ITM) who received 16 BCG-containing vaccines, and who after relapse received ICKi treatment without added toxicity.

## Methods

### Clinical Studies Update

The phase I study included patients with CM stages IIB, IIC, III, and IV (9). For the analysis here reported, patients at stage IV have been excluded. The Phase II CASVAC-0401 study included patients with CM stages IIB, IIC, and III (10). Both studies were carried out at the Instituto Alexander Fleming and were approved by the Ethics Committee of that institution. All patients signed informed consent following Helsinki protocols, and patient #5 (Pt#5) consented publication of her anonymized data.

### Immunohistochemistry

ITM biopsies from Pt#5 obtained before and after VACCIMEL immunization, and following nivolumab treatment, were formalin-fixed and paraffin embedded for histological analyses. From the latter ITM, a fresh punch biopsy was preserved at −80°C in RNAlater (Ambion) for RNA-seq analysis. Five-micrometer tumor sections were stained with anti-human monoclonal Abs to HLA- class I (A, B, C) (clone EMR8-5, Abcam), PMEL (clone hmb45, Dako), TYR (clone T311, Santa Cruz), CD8 (cloneC8/144, Dako), CD68 (clone PG-M1, Abcam), CD11c (clone EP1347Y, Abcam), DC-LAMP (clone 1010E1.01, Dendritics), and CD163 (clone 10D6, Invitrogen). The Avidin-Biotin-Peroxidase (ABC) system (Vectastain, Vector Labs) was used. Sections were examined by optical microscopy (Olympus BX40 microscope, DP2-BSW software). Quantification of the tumor-infiltrating immune populations in the different ITM biopsies was performed on the 1-mm^2^ hot spot area by duplicate (ImageJ software), distinguishing zones with peritumoral (PT) and intratumoral (IT) infiltrate. Immune cell counts in the different ITM biopsies were compared by Tukey’s multiple comparisons test; *p* < 0.05 was considered as statistically significant.

### RNA-Seq and Bioinformatic Analysis

Total RNA from frozen tumor was isolated with RNAaqueous Micro kit (Life Technologies); after DNase I treatment, RNA was quantified by a Nanodrop 1000 spectrophotometer (Thermo Fisher Scientific, Waltham, MA, USA). The RNA integrity number (RIN) algorithm was used to determine RNA quality on the Agilent 2100 Bioanalyzer using the Eukaryote Total RNA Nano kit (Agilent Technologies, Waldbronn, Germany). Finally, the generated libraries were sequenced on the Illumina HiSeq 2500 platform (Illumina, San Diego, CA, USA). In the bioinformatic analysis of the transcriptomic data, the FastQC software (18) was used to analyze the quality of raw reads. Sequencing reads were mapped on the human reference genome (GRCh38) using the TopHat2 software (19). The low-quality readings were removed with Picard Tools [Picard Tools By Broad Institute, available online at: http://broadinstitute.github.io/picard/ (accessed August28, 2020)] and the unmapped and non-properly paired reads were remapped using the BWA-MEM algorithm (20). Gene and isoform prediction were estimated using the Cufflinks method (21). The HTSeq software (v.0.6.0) (22) was used to calculate gene expression levels. The gene counts were normalized using the methods described in DESeq2 package (23). To explore the abundance of genes associated to the immune system, gene lists were collected and downloaded from two databases (24), the Immunology Database and Analysis Portal System (ImmPort) (https://immport.niaid.nih.gov) and InnateDB (http://www.innatedb.ca/). Manually curated gene subsets were also analyzed.

RNASeq data from the dermic metastasis was uploaded to the European Nucleotide Archive (ENA, EMBL EBI); the corresponding accession link is: https://www.ebi.ac.uk/ena/browser/view/PRJEB49671.

### IFNγ ELISPOT Assay

HLA haplotypes (class I and class II) were determined on PBMC isolated from pt#5 by Scisco Genetics 25, Nelson). Haplotype: HLA-A: 02:01:01/03:01:01; HLA-B 07:02:01/18:01:01; HLA-C: 07:01:01/07:02:01; DPA1: 01:03:01; DPB1: 04:01:01; DQA1: 01:02:01/05:05:01; DQB1: 03:01:01/06:02:01; DRB1: 11:04:01/15:01:01; DRB345: 02:02:01/01:01:01. We selected HLA-A*0201-restricted peptides corresponding to non-mutated PMEL, tyrosinase (TYR), MAGE-B2, and SOX-2 antigens, which were expressed in the vaccine cells, from the TANTIGEN DataBase (http://projects.met-hilab.org/tadb/). Selected peptides were either T-cell epitopes previously identified in functional assays (*in vitro* and/or *in vivo*) or HLA ligands as determined by physical detection (25). Tested peptides are detailed in **Table 1**. PBMC samples were thawed and seeded (1 × 10^6^) in 1 ml of CTL medium consisting of RPMI 1640 supplemented with 10% heat-inactivated human AB sera, 2 mM glutamine, 100 U/ml penicillin, 100 μg/ml streptomycin, 2.5 mM HEPES, and 50 U/ml IL-2, in 24-well plates. PBMCs were stimulated with peptides (10 μg/ml) and cultured at 37°C, in 5% CO_2_ for 12 days (effector cells). Every 3 days, fresh CTL medium with IL-2 was added. At day 10, additional PBMC samples were thawed, percentages of CD20+ and CD14+ cells (Ag-presenting cells, APCs) were recorded by flow cytometry, and cells were pulsed with peptides for 48 h. At day 12, APCs were treated with mitomycin C (50 μg/ml) for 1 h, washed twice with PBS, and resuspended in RPMI 1640 supplemented with FBS. Effector cells (4 × 10^4^) were seeded in 96-well plates previously coated with 5 μg/ml mouse anti-human IFNγ, and APCs were added in a 3:1 ratio (1.3 × 10^4^ CD20+ plus CD14+ cells/well) and cultured O.N. As a positive control, PBMCs (4 × 10^4^) were seeded and stimulated with 30 ng/ml OKT3 plus 1/1,000 PHA (M form, Gibco Life Technologies). As a negative control, non-stimulated cells were co-cultured with non-pulsed APC. Each experimental condition was performed in triplicate. Background baseline was calculated as the average number of spots present in non-stimulated cells for each time point sample (PRE, POST1, POST2, and POST3 samples, obtained at 0, 12, 18, and 25 months throughout VACCIMEL immunization). ELISPOT plates were developed as previously described (26). Plates were scanned using an AIDiSPOT ELR088IFL analyzer to quantify the number of spots per well; 350 spots/well were set as the maximum quantification limit.

**Table 1:** Peptides tested in ELISPOT assay for Patient#5.

Antigen	Peptide Sequence	Position in protein*	Haplotype restriction
PMEL/gp100 (1)	AMLGTHTMEV	177–186	A*02:01
PMEL/gp100 (2)	SLADTNSLAVV	570–580	A*02:01
TYROSINASE (1)	LLAVLYCLL	2–10	A*02:01
TYROSINASE (2)	LLWSFQTSA	9–17	A*02:01
SOX2 (1)	LLAPGGNSM	131–139	A*02:01
SOX2 (2)	SMYLPGAEV	275–283	A*02:01
MAGEB2	GVYDGEEHSV	231–240	A*02:01

*Amino acidic position.

### DMFS Analysis

We compared the distribution of DMFS of VACCIMEL-treated patients with those obtained by nivolumab (5), pembrolizumab (6), ipilimumab (27), and dabrafenib plus trametinib (28) treatments. Guyot’s iterative algorithm was applied to digitized published Kaplan–Meier curves to reconstruct individual patient data (29, 30). The digitization of source Kaplan–Meier curves was performed using WebPlotDigitizer (https://automeris.io/WebPlotDigitizer/). Survival analysis, including Kaplan–Meier analysis and Cox model fitting, was performed using R software (R Core Team 2020) using “survival” and “survminer” packages. A forest plot of hazard ratio was used to compare DMFS of different treatment vs. VACCIMEL. *p*-value< 0.05 was considered statistically significant.

## Results

### Clinical Update of VACCIMEL-Immunized Patients

**Figure 1:**
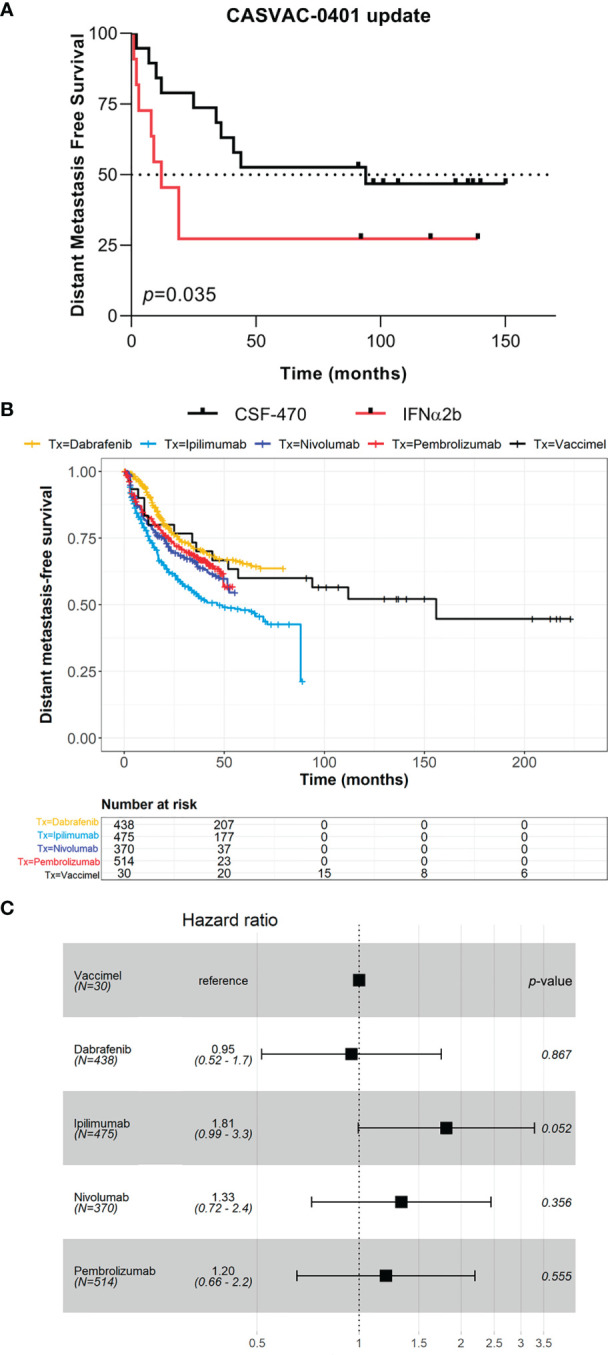
Clinical update of VACCIMEL-immunized patients. **(A)** DMFS follow-up of the CASVAC-0401 study patients as of 10/05/2021 (*p* = 0.035, Wilcoxon test). Arms: VACCIMEL (black) (*n* = 19); IFNα2b (red) (*n* = 11). **(B)** DMFS Kaplan–Meier and Cox-regression comparison of adjuvant treatments for CM patients. Treatments: Combined VACCIMEL-immunized patients from Phase I and II studies (black) (9, 10); dabrafenib (yellow) (28); ipilimumab (light blue) (27); nivolumab (blue) (5); pembrolizumab (red) (6). **(C)** Forest plot of hazard ratio comparison of DMFS of different treatments versus VACCIMEL.

The results of the Phase II study CASVAC-0401 demonstrated that vaccination with VACCIMEL plus BCG and GM-CSF was superior than medium-dose IFNα2b in terms of DMFS, with a minimum and maximum follow-up of 23 and 84 months, respectively (10). The CASVAC-0401 study was updated as of October 5, 2021, with a minimum and maximum follow-up of 91 months and 150 months (mean = 121 months). As represented by Kaplan–Meier analysis, DMFS was still significantly longer in vaccinated patients than in IFNα2b-treated patients (*p* = 0.035), with a median DMFS of 96 months for VACCIMEL and 13 months for IFNα2b (**Figure 1A**). The risk–benefit ratio of VACCIMEL remained unchanged. In order to analyze more patients with longer follow-up, we made a combined analysis of DMFS of vaccinated patients at stages IIB, IIC, and III recruited in the Phase I (9) and Phase II (10) studies (*n* = 30, **Table 2**). The mean age of the patients was 42.8 years, including 16 women (53%) and 14 men (47%). Clinical stages of all patients were re-determined as per version 8, AJCC (31). Of the 30 CM patients analyzed, 16/30 were at stage IIIC (53%); 9/30 were at stage IIIB (30%); 3/30 patients (10%) were at stage IIB; 1/30 (3%) was at stage IIIA; and 1/30 (3%) was at stage IIC. The minimum follow-up was 91 months and the maximum follow-up was 223 months. Median DMFS of the assembled population was 169 months. Also, overall survival (OS) data are shown (**Table 2**), but further analysis was not pursued (see under Discussion). We compared the DMFS of combined VACCIMEL data with other studies performed in similar populations of CM patients also treated in adjuvancy: ipilimumab versus placebo (4); pembrolizumab versus placebo (6); nivolumab versus ipilimumab (5); and dabrafenib/trametinib versus placebo (28) (**Figure 1B**). Analysis at 48 months follow-up between VACCIMEL and ipilimumab, nivolumab, pembrolizumab, and dabrafenib/trametinib, with Kaplan–Meier and Cox-regression models, did not attain statistically significant differences. Hazard ratio of the different treatments compared to VACCIMEL revealed no relevant statistically significant differences (**Figure 1C**). The only marginal significative difference (*p* = 0.052) was with ipilimumab presenting a hazard ratio of 1.81 versus VACCIMEL (VACCIMEL better). Therefore, a non-inferiority of VACCIMEL respect to the other treatments at 48 months follow up is suggested.

**Table 2:** Characteristics of patients vaccinated in Phase I and Phase II studies.

N	Sex	Age	Primary CM	N	AJCC stage	Clinical Study	DMFS	OS	Treatments post DM
1	M	42	T4b	N2b	IIIC	CASVAC	2	12	QT
2	M	52	T2a	N2b	IIIB	CASVAC	150+	150	0
3	F	33	T4b	N0	IIC	CASVAC	34	47	V
4	F	41	T3b	N2b	III C	CASVAC	141+	141+	0
5	F	33	T1b	N1c	IIIB	CASVAC	136+	136+	V, HP, N, D/TRA
6	M	61	T0	N2b	IIIC	CASVAC	137+	137+	0
7	M	48	T4b	N1a	III C	CASVAC	94	120	RT; N; D/Tra
8	M	50	T3a	N1a	III B	CASVAC	130+	130+	0
9	F	51	T3a	N1a	IIIB	CASVAC	25	79	V
10	F	39	T2a	N2a	IIIB	CASVAC	12	30	V
11	M	47	T3a	N1a	III B	CASVAC	107+	107+	0
12	M	46	T3B	N2A	IIIC	CASVAC	57	89+	U
13	M	35	T3a	N1a	III B	CASVAC	101+	101+	0
14	M	40	T3b	N3	IIIC	CASVAC	10	14	RT; V
15	F	51	T4b	N1a	IIIC	CASVAC	97+	97+	0
16	M	35	T3b	N1b	IIIC	CASVAC	7	14	0
17	F	61	T4a	N0	IIB	CASVAC	36	77+	U
18	F	43	T2a	N2c	IIIC	CASVAC	91+	91+	0
19	M	46	T4b	N0	IIC	CASVAC	44	91+	N
20	F	49	T?	N1	IIIA	VACCIMEL	216+	216+	0
21	M	33	T2	N0	IIB	VACCIMEL	52	90	U
22	F	45	T2a	N0	IIB	VACCIMEL	204+	204+	0
23	F	33	T0	N3b	IIIC	VACCIMEL	112	132	I
24	F	15	T4a	N2b	IIIC	VACCIMEL	218+	218+	0
25	F	56	T0	N2b	IIIC	VACCIMEL	213+	213+	0
26	F	32	T2b	N1b	IIIB	VACCIMEL	223+	223+	0
27	F	48	T0	N2b	IIIC	VACCIMEL	218+	218+	0
28	M	28	T2b	N1c	IIIC	VACCIMEL	3	28	QT
29	M	65	T4a	N1c	IIIC	VACCIMEL	156	156	U
30	F	65	T4a	N1c	IIIC	VACCIMEL	10	23	QT

Clinical stages were determined according to 8th edition AJCC. DMFS, distant metastasis-free survival; OS, overall survival; DM, distant metastases; 0, no treatment; HT, hyperthermic perfusion; QT, chemotherapy; V, vemurafenib; I, ipilimumab; RT, radiotherapy; N, nivolumab; D/Tra, dabrafenib/trametinib; U, unknown.

### Compatibility of VACCIMEL Plus BCG Treatment With Ulterior Immunotherapy

Based on studies performed in *Macacus rhesus* that suggested temporary reactivation of chronic SIV infections with anti-PD-1 treatment, clinical assays performed with anti-PD-1 mAbs precluded the use of live-attenuated vaccines during anti-PD-1 therapy (15, 16). The information about the effect of anti-PD-1 mAbs on tuberculosis evolution is conflicting, with evidence suggesting awakening of infection (32, 33), while other authors (34) suggest the opposite effect. Therefore, our hypothesis that vaccination expands lymphocytic clones that could be further amplified with anti-PD-1 treatment required previously answering the important question if treatment with VACCIMEL, which includes as adjuvants BCG and GM-CSF, induced BCG reactivation and infection after anti-PD-1 treatment. Three patients from our clinical assay CASVAC-0401 (#5, #7, and #19) (**Table 2**) who after ending the CASVAC-0401 protocol progressed to metastatic disease and were treated with nivolumab achieved complete responses without any signs of toxicity due to BCG infection (JM, personal communication). As an example, Pt #5 shall be described in detail, since this patient received 16 i.d. vaccine doses during treatment and follow-up. Due to disease progression after VACCIMEL treatment, the patient underwent various treatments, including 4 years of anti-PD-1 treatment. Besides, due to her ITM disease, several biopsies could be obtained along her treatment.

**Figure 2:**
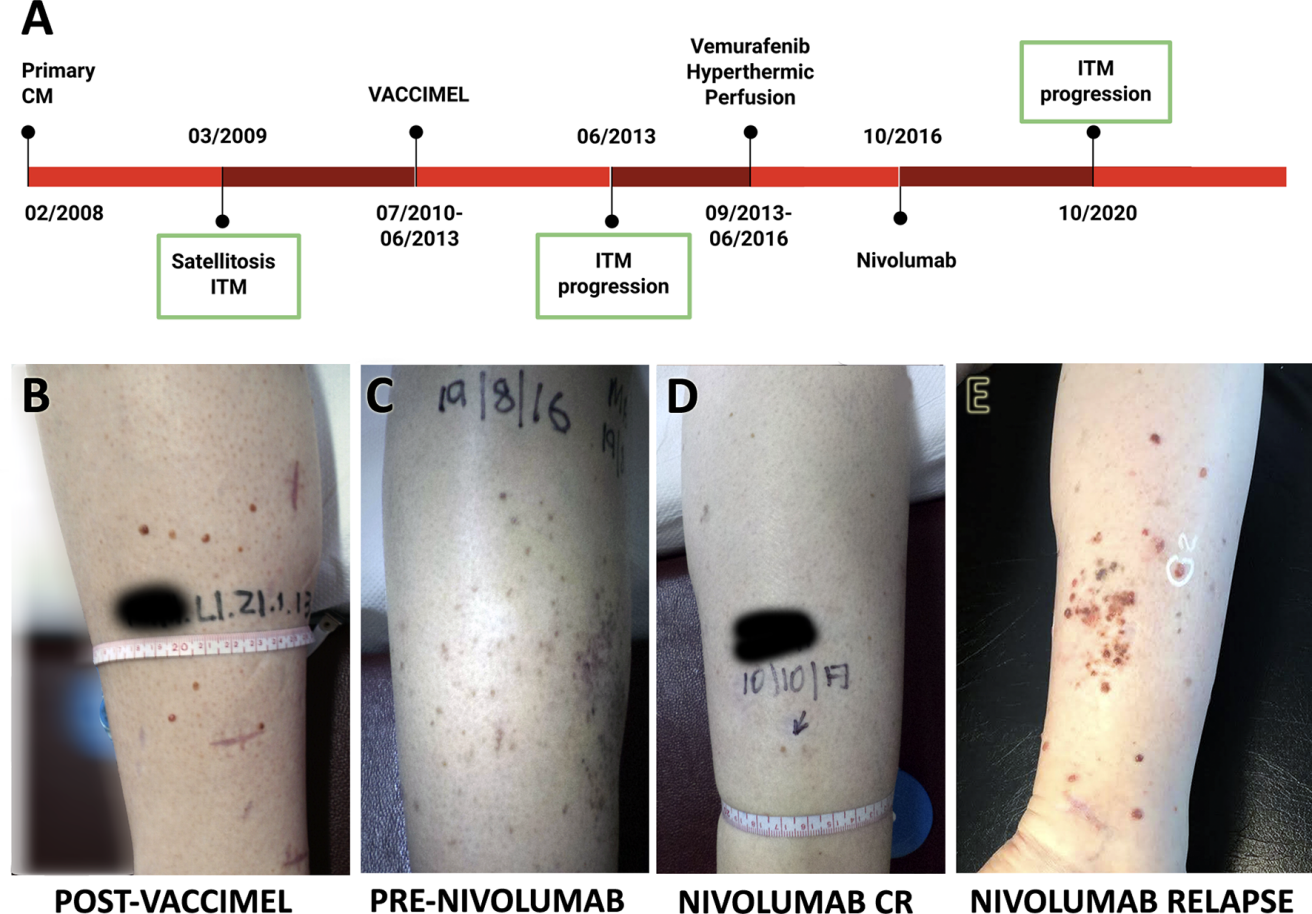
Patient #5 case report. **(A)** Time course of the disease and treatments received. ITM biopsies analyzed are indicated with a green box. **(B–E)** Pictures of ITM on patient’s right leg: **(B)** relapse after ending vaccination protocol; **(C)** progression before nivolumab; **(D)** response after nivolumab 240 mg every 14 days for 1 year; **(E)** relapse after 4 years nivolumab, showing multiple red amelanotic 1- to 3-mm-wide tumors.

The timeline of Pt#5 clinical evolution with most relevant events is shown in **Figure 2A**. She is a 44-year-old female who in 02/2008 presented in her right leg a superficial extensive melanoma, Breslow 0.89 mm, ulcerated, in vertical growth phase, and with brisk lymphocyte infiltration. Margins were enlarged and two sentinel node biopsies were negative for metastasis. In 03/2009, cutaneous satellites appeared, progressing into ITM, many of which were removed. On 07/2010, the patient entered the CASVAC-0401 Phase II protocol in the vaccine arm and completed the 2-year protocol in 08/2012, after which she received three more vaccinations. The cutaneous lesions, mostly located at the inner part of her right leg, which had remained stable until then, progressively began to increase in number and size from 06/2013 (**Figure 2B**). Biopsies documented melanoma metastases, positive for BRAF^V600E^ oncogene mutation, and PET/CT revealed no distant metastasis. Given the impossibility of complete tumor removal (Stage IIIB), she began treatment with vemurafenib 960 mg BID, with complete clinical response until 2015, when ITM relapsed without any sign of distant metastasis by PET/CT. Two cycles of hyperthermic perfusion with melphalan were performed, and flattening of the lesions without complete resolution was observed After recurrence of the lesions in 2016 (**Figure 2C**), mainly at the posterior part of the leg, nivolumab (240 mg every 14 days) was started with an excellent initial response, showing almost disappearance of the lesions and good tolerance (**Figure 2D**). Four years later, regional ITM reappeared in large numbers, as multiple 1- to 3-mm amelanotic tumors at the back of the leg, and nivolumab was interrupted (**Figure 2E**). As confirmed by CAT and PET-CAT scans, the disease continued confined to the right leg and no lung BCG nodules were observed. Two punch biopsies were obtained at 10/2020 for histology, immunohistochemistry, and RNA-seq analysis. Pt#5 started treatment with dabrafenib/trametinib (12/2020) that continues until the writing of this paper with stable disease. Histology and immunostaining comparative analysis were performed on ITM biopsies obtained before and after VACCIMEL immunization, along with biopsies after nivolumab treatment (**Figure 2A**), of which a fresh punch biopsy was obtained for RNA-seq analysis.

**Figure 3:**
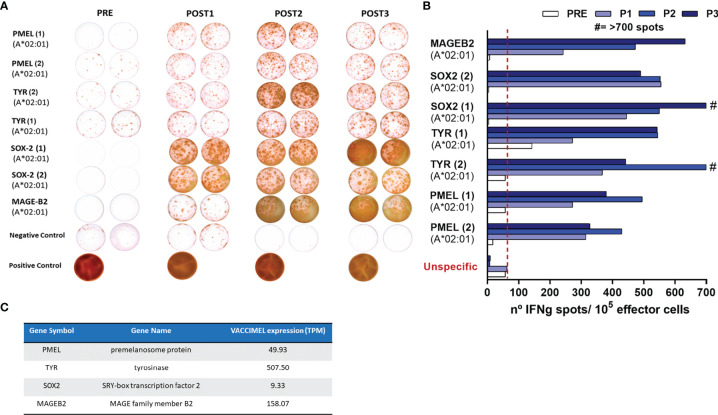
Patient #5 immune response to common melanoma antigens. **(A)** T-cell response induced by VACCIMEL to HLA-A0201-restricted peptides from shared melanoma-associated antigens detected by IFN-γ ELISPOT. Pre: blood extracted at the selection process; Post 1, Post 2, and Post 3: blood extracted 6, 12, and 25 months after protocol start. **(B)** Quantification of the spots normalized to 10^5^ PBMC (right panel). **(C)** PMEL, tyrosinase (TYR), SOX-2 and MAGE B2 antigens abundance in VACCIMEL as determined by RNA-Seq and normalized in TPM.

**Figure 4:**
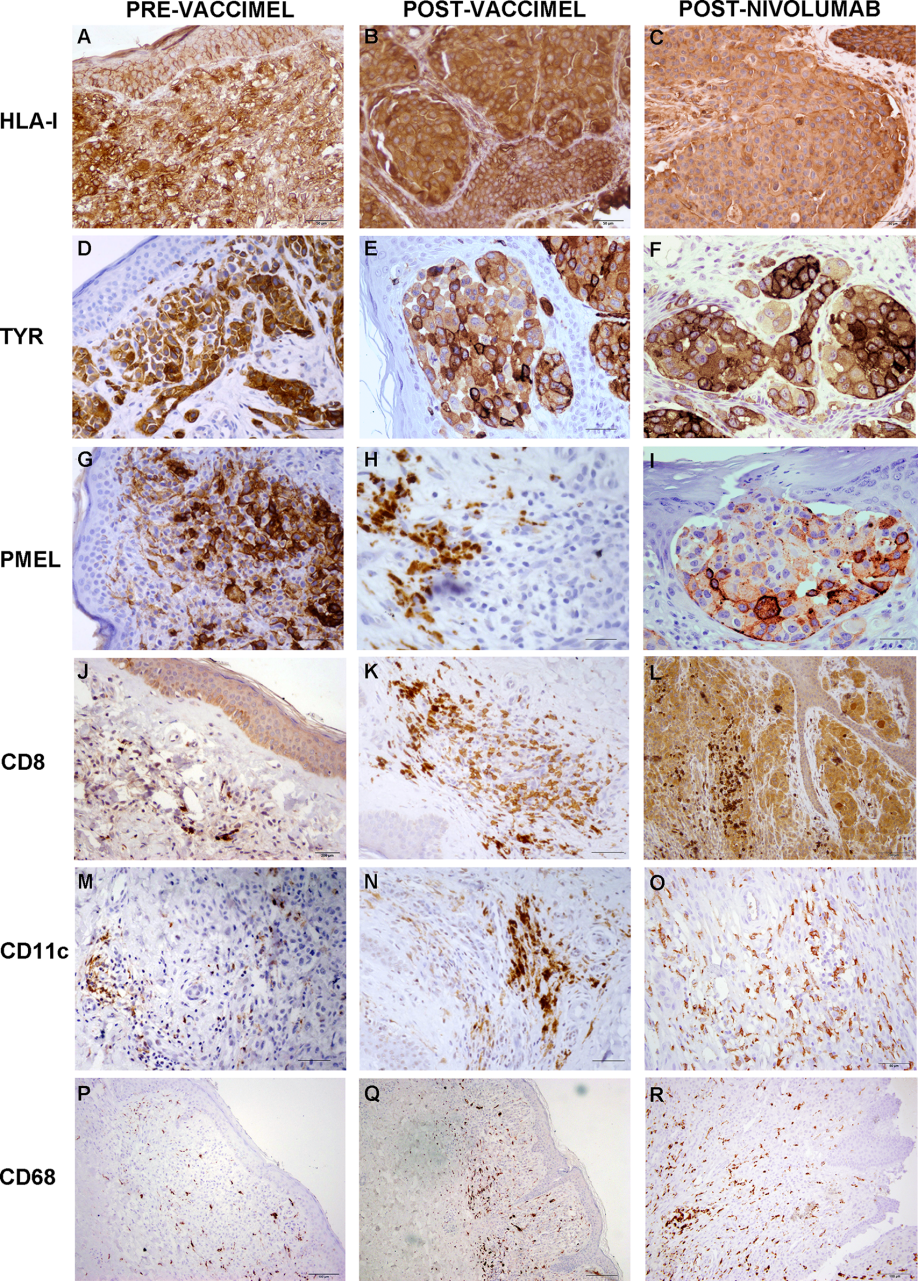
Comparative analysis of Patient#5 ITM immune populations. Immunostaining was performed as described under Methods on ITM biopsies obtained before VACCIMEL immunization, after VACCIMEL immunization, and following nivolumab treatment: **(A–C)** HLA class I; **(D–F)** TYR; **(G–I)** PMEL; **(J–L)** CD8; **(M–O)** CD11c; **(P–R)** CD68. Scale bars: **(A–I)** 50 µm; **(J–O)** 100 µm; **(P–R)** 200 µm.

In order to assert if VACCIMEL plus BCG elicited toxicity after posterior anti-PD-1 treatment, immune proficiency of Pt #5 had to be previously demonstrated. The frequency of T-cell clones recognizing synthetic peptides of shared TAA was determined by ELISPOT in PBMC samples obtained throughout the CASVAC-0401 protocol (PRE, 6, 12, and 25 months). VACCIMEL induced expansion of a T-cell response to HLA-A0201-restricted peptides from shared non-mutated melanoma antigens (**Figures 3A, B**). It is important to note that reactivity was practically nil before vaccination (PRE sample), although PMEL and tyrosinase (TYR) were strongly expressed in the patient’s tumor (**Figure 4**). For most of the assayed peptides, a clear increase in the immune response was observed during the 2-year vaccination protocol. It should be noted that the intensity of the immune response towards the different antigens appeared unrelated to their expression abundancy in the vaccine or in the tumor (**Figure 3C**). Thus, patient’s immune response to a low-expressing gene as SOX2 in the vaccine was equally strong as the response to highly expressed genes as PMEL and TYR.

**Figure 5:**
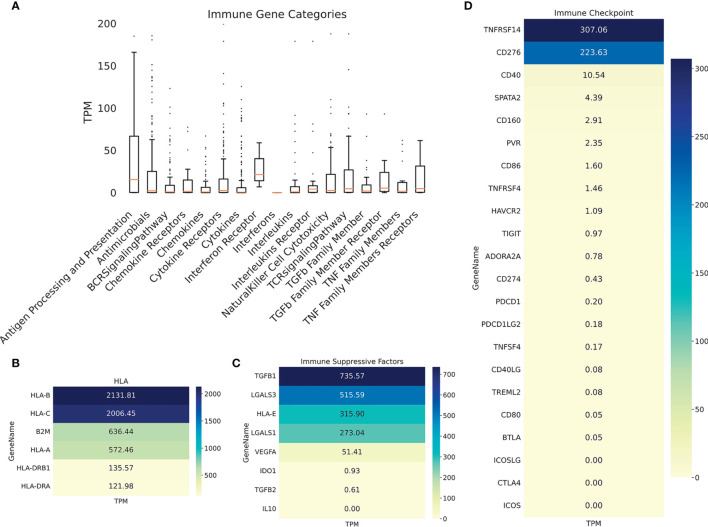
Patient #5 ITM immune genes expression profiling. **(A)** Boxplot showing expression of immune genes grouped in multiple categories, as described under Methods. Heatmaps showing expression of selected genes: **(B)** HLA molecules, **(C)** immunosuppressive molecules; **(D)** immune checkpoints (receptors and ligands).

Immunohistochemistry was performed on PRE- and POST-vaccination biopsies, and of that obtained after nivolumab recurrence, to determine if any changes in the expression of relevant TAA and immune cells occurred during the patient’s evolution (**Figure 4**). HLA expression was maintained all along the disease evolution (**Figures 4A–C**), and the expression of TAA gp100/Pmel and tyrosinase/Tyr was also abundant (**Figures 4D–I**). The number of CD8^+^ lymphocytes, CD11c^+^ antigen-presenting cells, and CD68^+^ macrophages increased following successive treatments (**Figures 4J–R** and **Supplementary Figure 1**). In addition, the biopsy obtained after nivolumab relapse was analyzed by RNASeq. We searched in this unique sample the expression of genes related to the immune system. The expression of genes involved in antigen processing and presentation was high, as well as TCR Signaling, IFN, TNF, and TGFβ receptors (**Figure 5A**). Expression of HLA class I and class II molecules as well as of β2M was abundant (**Figure 5B**). Also, several immunosuppressive molecules such as TGFB1, HLA-E, and galectins 1 and 3 were abundant; no IL-10 expression was detected (**Figure 5C**). Immune checkpoints (receptors and ligands) such as CTLA-4, PD-1 (PDCD1), and PD-L1 (CD274) had low expression levels in this post-treatment biopsy (**Figure 5D**). These results pointed to a coexistence in the tumor of immunostimulatory and immunosuppressive characteristics.

## Discussion

We shall first discuss the updated adjuvant CASVAC-0401 study that has now a maximum follow-up of 150 months. We have chosen DMFS as an end point for this study, since OS is not reliable because our patients have been recruited over a long period of time and different treatments have become available over the years and may confound the final results. Besides, DMFS is a biological meaningful end point, since loco-regional relapses do not alter much final survival (35). The DMFS in the CASVAC-0401 study continues to be significantly higher (*p* = 0.035) for the vaccine arm versus IFNα2b treatment (median DMFS 96 months versus 13 months, respectively). Since two patients (#7 and #12) progressed to distant metastases at 94 and 53 months, respectively, after entering the protocol, and in order to analyze more vaccinated patients with a longer follow-up, we combined patients treated in our Phase I study [stages IIB (*n* = 2) and III (*n* = 9)], who had much longer follow-up, with those of the Phase II study, in a single cohort. The combined analysis of DMFS included 30 patients with CM at stages IIB, IIC, IIIA, IIIB, and IIIC (3:1:1.9:16) treated with VACCIMEL, with a follow-up attaining approximately 20 years (**Table 2**). The DMFS of patients treated with VACCIMEL was 71.4% at 48 months, and median DMFS was reached at 169 months. As shown under Results, the hazard ratio for VACCIMEL as compared to adjuvant nivolumab, pembrolizumab, ipilimumab, and dabrafenib/trametinib, in which results updated to 48 months follow-up were published, did not attain statistical significance. Even taking into account the different number of patients included, our results are not inferior to those of Ascierto et al. (5) in which they assayed nivolumab versus ipilimumab in resected stage IIIA, IIIB, IIIC, and IV CM patients. After 48 months of follow-up, the rate of DMFS in the stage III ITT population was 59.2% for nivolumab and 53.3% for ipilimumab. Also, Eggermont et al. (6) analyzed DMFS in stage III CM patients treated in adjuvancy with pembrolizumab versus placebo. After 48 months of follow-up, DMFS was 63% for pembrolizumab and 49.5% for placebo. It should be noted that in Stage IIIA patients, the benefit of pembrolizumab versus placebo did not attain statistical significance. In another study in which ipilimumab versus placebo was analyzed (27), DMFS at 48 months was 50.8% for ipilimumab versus 41.7% for placebo. In a BRAF-mutated cohort of stage III CM patients, Dummer et al. reported, after 48 months follow-up, that 67% of patients survived free of distant metastases versus 56% of placebo-treated patients (28). In a real-life study, De Meza et al. (36) reported that 61% of the CM patients treated in adjuvancy with pembrolizumab or nivolumab had to interrupt their treatment due to adverse effects. In contrast, none of the VACCIMEL-treated patients dropped out of the study due to toxicity (9, 10). All in all, it may be concluded that when the stage III CM population is treated in adjuvancy with immunotherapy or with dabrafenib/trametinib in a BRAF^V600^ mutated population, only approximately 10%–15% of patients shall benefit from treatment, since approximately 50% of patients will not recur even without treatment, and approximately 35%–40% of patients shall not respond. Therefore, more molecular insight is needed to discern between those populations and great efforts are being done on treatment combinations.

Patient #5 is described here since (i) she participated in the vaccine arm of the CASVAC-0401 trial and received 16 BCG-containing vaccines as part of her treatment, and (ii) she developed strong immune response to vaccination. After progression, and among other treatments, the patient was treated between 2016 and 2020 with nivolumab. It is interesting to note that previous treatment with VACCIMEL did not trigger any untoward adverse effects to ICKi treatment, thus suggesting that sequential combinations of both treatments is feasible. With respect to the possible incompatibility between BCG and anti-PD-1 antibodies, the previous evidence is conflicting. On one hand, Kaufman et al. (32) found that anti-PD-1 favored the virulent *Mycobacterium tuberculosis* growth in rhesus macaques, probably due to increased inflammation in granulomas. Also, Barber et al. (33) found that in two cancer patients treated with anti-PD-1, disseminated TBC was triggered. Even though Th1 cells through IFNγ and TNFα are required for host control of mycobacteria, their enhanced activity in anti-PD-1 blockade seems to worsen infection in some cases. On the contrary, Sakai et al. (34) analyzed BCG infection in mice and found that the axis PD-1/PDL-1 impaired Th1 immunity and favored BCG persistence, proposing anti-PD-1 mAbs as a potential treatment for latent TB or vaccination enhancement therapy. It is plausible that the response to anti-PD-1 mAbs depends on the treatment timing and the particular microbe present, behaving differently whether it is *M. tuberculosis* or BCG, an attenuated vaccine strain. It should be stressed that vaccination of newborns with BCG is mandatory in Argentina.

Immune analysis of this patient demonstrated that (i) reactive clones of lymphocytes specific for TAA, non-existing before vaccination, were induced during the study, thus confirming and expanding previous results; (ii) a tumor biopsy was obtained after 4 years of treatment with nivolumab, and RNA-seq and immunohistochemical studies were performed. These analyses demonstrated the co-existence of immune stimulation, basically in peripheral mononuclear cells and at the tumor, with CD8 T cells and CD11c+ antigen-presenting cells infiltrating the lesions, as well as an immunosuppressive TME, characterized by high expression of TGFβ1 and other immune-suppressive molecules. The opposite forces between reactive lymphocytic clones against shared TAA and an immunosuppressive TME may perhaps explain why the patient only temporarily responded and progressed to alternative treatments of VACCIMEL and ICKi, although the disease was confined to her right leg for more than 11 years and she had an excellent quality of life.

Therefore, we conclude that VACCIMEL in adjuvancy prolongs DMFS in stages IIB, IIC, and III CM patients. Its mechanism of action would be to increase the number of reactive lymphocytic clones against TAA and neoAgs. Besides its own efficacy, we suggest that treatment with VACCIMEL could prime further immunotherapies without additional toxic effects.

## Data Availability Statement

The datasets presented in this study can be found in online repositories. The names of the repository/repositories and accession number(s) can be found at: https://www.ebi.ac.uk/ena, PRJEB49671.

## Ethics Statement

The studies involving human participants were reviewed and approved by Instituto Alexander Fleming Ethics Committee (Buenos Aires, Argentina). The patients/participants provided their written informed consent to participate in this study.

## Author Contributions

MB and JM: conception and design, collection and assembly of data, data analysis and interpretation, and manuscript writing. They are Sub/Principal Investigator of the CASVAC-0401 study. AM, MA, IC, JP, EP, and AB: collection and assembly of data, data analysis and interpretation, and manuscript writing. AM and MA contributed equally to the study. GC: statistical analysis. All authors contributed to the article and approved the submitted version.

## Funding

This work was supported by grants from CONICET, Instituto Nacional del Cáncer—Ministerio de Salud de la Nación Argentina (INC-MSal) INC III, IS001946, Fundación Sales, Fundación Cáncer, Fundación Pedro F. Mosoteguy, Argentina. The CASVAC-0401 clinical study was sponsored by Laboratorio Pablo Cassará S.R.L.

## Conflict of Interest

The authors declare that the research was conducted in the absence of any commercial or financial relationships that could be construed as a potential conflict of interest.

## Publisher’s Note

All claims expressed in this article are solely those of the authors and do not necessarily represent those of their affiliated organizations, or those of the publisher, the editors and the reviewers. Any product that may be evaluated in this article, or claim that may be made by its manufacturer, is not guaranteed or endorsed by the publisher.
