# Supplementary material for: An Update of Cutaneous Melanoma Patients Treated in Adjuvancy With the Allogeneic Melanoma Vaccine VACCIMEL and Presentation of a Selected Case Report With In-Transit Metastases
Source: Front Immunol. 2022 Apr 1;13:842555. doi: 10.3389/fimmu.2022.842555 (PMC9011367; doi:10.3389/fimmu.2022.842555)
Supplement: Supplementary file 1 [file Image_1.pdf]

A

Immune populations	N° cells/mm^2		
	**PRE-VACCIMEL**	**POST-VACCIMEL**	**POST-NIVOLUMAB**
CD8 PT	147 ± 8	378 ± 11	706 ± 10
CD8 IT	1 ± 0	48 ± 8	106 ± 5
CD11c PT	42 ± 3	198 ± 6	207 ± 8
CD11c IT	19 ± 2	114 ± 3	205 ± 9
CD68 PT	96 ± 7	274 ± 9	150 ± 3
CD68 IT	104 ± 4	244 ± 5	137 ± 8

PT: peri-tumoral, IT: intra-tumoral

B

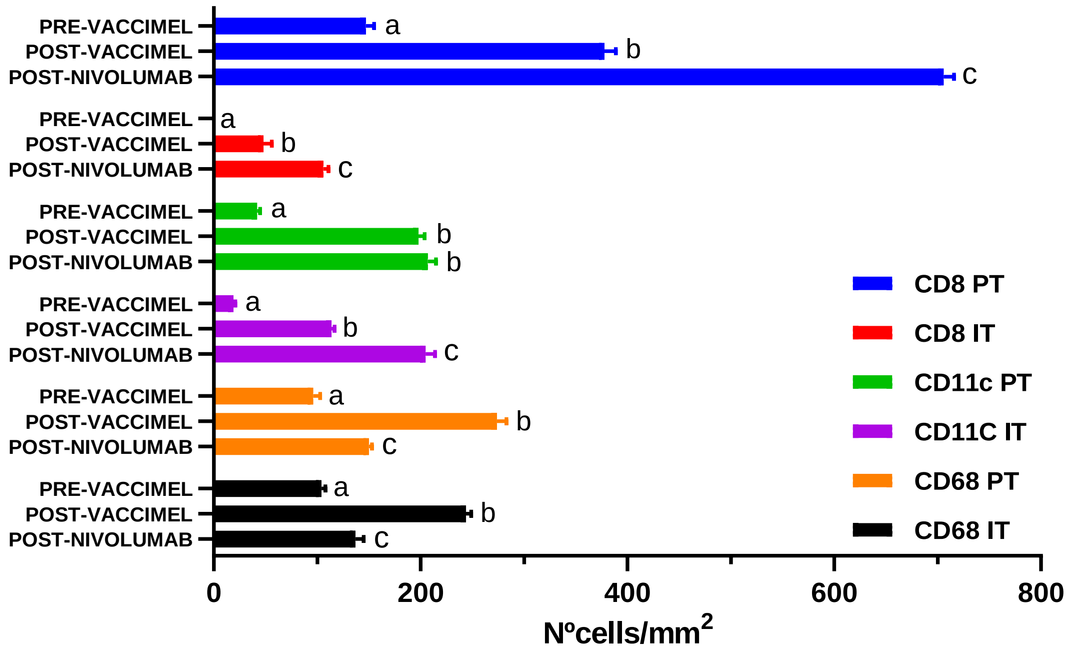

Supplementary Figure 1. Immune infiltrate analysis of Patient#5 ITM biopsies. Comparative analysis of tumor-infiltrating immune populations was performed by immunohistochemistry on ITM biopsies obtained before and after VACCIMEL immunization, and following nivolumab treatment, as described under Methods. **(A)** Table with quantification of CD8, CD11c and CD68 positive cells, distinguishing zones with peritumoral (PT) and intratumoral (IT) infiltrate. **(B)** Bars show mean cells/mm^2 ±SD; bars with different letters are statistically different (Tukey's multiple comparisons test, $p < 0.05$)
